# Inhibition of Nigrostriatal Dopamine Release by Striatal GABA_A_ and GABA_B_ Receptors

**DOI:** 10.1523/JNEUROSCI.2028-18.2018

**Published:** 2019-02-06

**Authors:** Emanuel F. Lopes, Bradley M. Roberts, Ruth E. Siddorn, Michael A. Clements, Stephanie J. Cragg

**Affiliations:** ^1^Centre for Integrative Neuroscience, Department of Physiology, Anatomy and Genetics, University of Oxford, Oxford OX1 3PT, United Kingdom, and; ^2^Oxford Parkinson's Disease Centre, Oxford OX1 3PT, United Kingdom

**Keywords:** dopamine, GABA, presynaptic, striatum, voltammetry

## Abstract

Nigrostriatal dopamine (DA) is critical to action selection and learning. Axonal DA release is locally influenced by striatal neurotransmitters. Striatal neurons are principally GABAergic projection neurons and interneurons, and a small minority of other neurons are cholinergic interneurons (ChIs). ChIs strongly gate striatal DA release via nicotinic receptors (nAChRs) identified on DA axons. Striatal GABA is thought to modulate DA, but GABA receptors have not been documented conclusively on DA axons. However, ChIs express GABA receptors and are therefore candidates for potential mediators of GABA regulation of DA. We addressed whether striatal GABA and its receptors can modulate DA release directly, independently from ChI regulation, by detecting DA in striatal slices from male mice using fast-scan cyclic voltammetry in the absence of nAChR activation. DA release evoked by single electrical pulses in the presence of the nAChR antagonist dihydro-β-erythroidine was reduced by GABA or agonists of GABA_A_ or GABA_B_ receptors, with effects prevented by selective GABA receptor antagonists. GABA agonists slightly modified the frequency sensitivity of DA release during short stimulus trains. GABA agonists also suppressed DA release evoked by optogenetic stimulation of DA axons. Furthermore, antagonists of GABA_A_ and GABA_B_ receptors together, or GABA_B_ receptors alone, significantly enhanced DA release evoked by either optogenetic or electrical stimuli. These results indicate that striatal GABA can inhibit DA release through GABA_A_ and GABA_B_ receptors and that these actions are not mediated by cholinergic circuits. Furthermore, these data reveal that there is a tonic inhibition of DA release by striatal GABA operating through predominantly GABA_B_ receptors.

**SIGNIFICANCE STATEMENT** The principal inhibitory transmitter in the mammalian striatum, GABA, is thought to modulate striatal dopamine (DA) release, but definitive evidence for GABA receptors on DA axons is lacking. Striatal cholinergic interneurons regulate DA release via axonal nicotinic receptors (nAChRs) and also express GABA receptors, but they have not been eliminated as potentially critical mediators of DA regulation by GABA. Here, we found that GABA_A_ and GABA_B_ receptors inhibit DA release without requiring cholinergic interneurons. Furthermore, ambient levels of GABA inhibited DA release predominantly through GABA_B_ receptors. These findings provide further support for direct inhibition of DA release by GABA receptors and reveal that striatal GABA operates a tonic inhibition on DA output that could critically influence striatal output.

## Introduction

The striatum plays key roles in promoting motivated behaviors and learned actions. Nigrostriatal dopamine (DA) neurons release DA from immensely arborized structures, with each neuron forming ∼10^5^ en passant varicosities and reaching ∼2.7% of striatum in rat (Matsuda et al., 2009). DA output is gated by numerous striatal neuromodulators (Sulzer et al., 2016). Cholinergic interneurons (ChIs) play a particularly powerful role in gating and driving DA release through nicotinic acetylcholine receptors (nAChRs) on DA axons (Jones et al., 2001; Zhou et al., 2001; Rice and Cragg, 2004; Zhang and Sulzer, 2004; Threlfell et al., 2012). ChIs make up 1–2% of striatal neurons (Oorschot, 1996), with the remaining 98% being GABAergic. Striatal GABA neurons include the principal spiny project neurons (SPNs) (∼95%) as well as interneurons (∼2–3%) including fast-spiking interneurons (FSIs) and low-threshold spiking interneurons (LTS), among others (Gittis and Kreitzer, 2012). Furthermore, DA axons might provide a source of co-released GABA (Tritsch et al., 2012, 2014; Kim et al., 2015). Striatal GABA_A_ and GABA_B_ receptors have been shown to modulate DA release, but information about their localization to DA axons is lacking. A role for ChIs as potential mediators of GABA regulation of DA has not been excluded.

Striatal administration of pregnanolone, a positive allosteric modulator of the GABA_A_ receptor, or bicuculline, a GABA_A_ antagonist, respectively decreased and increased extracellular dopamine in intact rats measured by microdialysis (Smolders et al., 1995) and muscimol, a GABA_A_ agonist, inhibited DA release from striatal synaptosomes (Ronken et al., 1993). A GABA_A_-mediated enhancement of DA release has been reported in guinea pig striatal slices during prolonged electrical pulse trains, but this is thought to arise indirectly via inhibition of H_2_O_2_ release from striatal neurons during prolonged stimuli (Avshalumov et al., 2003). Striatal perfusion of the GABA_B_ agonist baclofen or the antagonist phaclofen respectively decreased and increased extracellular dopamine in intact rats (Smolders et al., 1995) and baclofen decreased electrically evoked DA release in acute slices of mouse caudate putamen (Schmitz et al., 2002) and nucleus accumbens (Pitman et al., 2014). Findings in slice preparations are less confounded by potential effects on long loop circuits *in vivo* that could regulate DA via changes in DA neuron firing and therefore more directly support a local mechanism of action. However, whereas both GABA_A_ and GABA_B_ receptors are densely expressed throughout striatum (Ng and Yung, 2000; Waldvogel et al., 2004), only the GABA_B_ receptor has been indicated on structures that resemble DA axons. Ultrastructural studies report GABA_B_ receptors in striatal neuropil in monkey and rat (Charara et al., 2000; Yung et al., 1999), which is consistent with, but not definitive evidence for, GABA_B_ receptors on DA axons.

Both GABA_A_ and GABA_B_ receptors are present on ChIs (Waldvogel et al., 1998; Yung et al., 1999). Because ChIs operate strong control over DA release and can mediate effects of other neuromodulators on DA, including opioids, nitric oxide, glutamate, and insulin (Britt and McGehee, 2008; Hartung et al., 2011; Stouffer et al., 2015; Kosillo et al., 2016), ChIs emerge as the potential mediators of GABAergic inhibition of DA release. ChIs need to be excluded as potential mediators before direct regulation of DA by GABA_A_ and/or GABA_B_ receptors seems more plausible.

Here, we explored the control of DA release by GABA, GABA_A_ receptors, and GABA_B_ receptors in the absence of ACh input to nAChRs. We assessed GABA receptor regulation of DA release when evoked electrically in the presence of nAChR antagonist dihydro-β-erythroidine (DHβE) and also when DA release was evoked by targeted activation of DA axons using optogenetics when DA release is not under nAChR control (Threlfell et al., 2012; Melchior et al., 2015). We found that GABA_A_ and GABA_B_ receptors can inhibit DA release independently from nAChR activation, providing further support for a direct mechanism of action. Furthermore, we found that endogenous striatal GABA provides a tonic inhibition of DA release.

## Materials and Methods

### 

#### 

##### Animal preparation and surgery.

Animals were either wild-type C57BL6 male mice at postnatal day 35 (P35) to P70 (RRID:IMSR_JAX:000664) or DAT-Cre mice injected with an adeno-associated virus encoding Cre-dependent ChR2. For experiments with light activation, DAT-Cre mice were bred from homozygotes for DAT-internal ribosome entry site (IRES)-Cre, obtained from Jackson Laboratories (B6.SJL-*Slc6a3*^tm1.1(cre)Bkmn^ /J, stock 006660) (RRID:IMSR_JAX:006660). P25–P35 DAT-Cre mice were anesthetized with isoflurane, placed in a small animal stereotaxic frame (David Kopf Instruments), and injected with an adeno-associated virus (∼10^12^ genome copies per milliliter; UNC Vector Core Facility, Chapel Hill, NC) encoding Cre-dependent ChR2 (AAV5-EF1α-DIO-hChR2(H134R)-eYFP) (RRID:SCR_002448). A total volume of 1 μl of virus solution was injected bilaterally (500 nl per hemisphere/injection) into substantia nigra pars compacta (SNc, AP −3.1 mm, ML ±1.2 mm from bregma, DV −4.25 mm from exposed dura mater). Virus solution was injected at an infusion rate of 50 nl/min with a 32 gauge Hamilton syringe and withdrawn 5–10 min after the end of injection. Virus-injected mice were used for experiments >4 weeks after viral injection.

##### Slice preparation.

Wild-type C57BL6 animals (P35–P70) or DAT-Cre mice (P53–P63) were decapitated after cervical dislocation and the brains were extracted. Coronal slices, 300 μm thick, were prepared in ice-cold HEPES-buffered artificial CSF (aCSF) containing the following (in mm): 120 NaCl, 20 NaHCO_3_, 6.7 HEPES acid, 5 KCl, 3.3 HEPES salt, 2 CaCl_2_, 2 MgSO_4_, 1.2 KH_2_PO_4_, and 10 glucose. Slices were then maintained in HEPES-buffered artificial CSF at room temperature for at least 1 h before recording. All procedures were performed according to institutional guidelines and conformed to the UK Animals (Scientific Procedures) Act of 1986.

##### Fast-scan cyclic voltammetry.

Evoked DA release was detected in acute slices using fast-scan cyclic voltammetry (FCV). Slices were superfused with a bicarbonate-buffered aCSF saturated with 95% O_2_/ 5% CO_2_ at 31–32°C containing the following (in mm): 124 NaCl, 26 NaHCO_3_, 3.8 KCl, 2.4 CaCl_2_, 1.3 MgSO_4_, 1.3 KH_2_PO_4_, and 10 glucose. All experiments with electrical stimulation were conducted in the presence of 1 μm DHβE to prevent the effects of nAChR activation on DA release (Zhou et al., 2001; Rice and Cragg, 2004). Extracellular DA concentration ([DA]_o_) was monitored using FCV with 7-μm-diameter carbon fiber microelectrodes (tip length 50–100 μm) and a Millar voltammeter (Julian Millar, Barts and the London School of Medicine and Dentistry) as described previously (Threlfell et al., 2010). In brief, the scanning voltage was a triangular waveform (−0.7 V to +1.3 V range versus Ag/AgCl) at a scan rate of 800 V/s and sampling frequency of 8 Hz. Signals were attributable to DA by the potentials for peak oxidation and reduction currents (oxidation peak: +500–600 mV, reduction peak: ∼−200 mV). Electrodes were calibrated *post hoc* with 2 μm DA in experimental medium. None of the drugs altered electrode sensitivity to DA. Data were acquired and analyzed using Axoscope 10.6 (Molecular Devices) and locally written Excel macros.

##### Electrical and light stimulation.

Recordings were obtained from the dorsal striatum. Electrical stimulation was delivered by a local bipolar concentric electrode (25 μm diameter, Pt/Ir; FHC). All experiments with electrical stimulation were conducted in the presence of 1 μm DHβE, which prevents nAChR activation. Stimulation intensity was set to 80% maximal [DA]_o_: ∼0.6 mA. Applied stimuli were single 200 μs pulses (1p) or five pulses (5p) at 5, 25, and 100 Hz. Mean peak [DA]_o_ evoked by 1p was equivalent to that of a 1 Hz train; 1p is used in frequency comparison to indicate maximum 1 Hz data. Electrical stimulations were repeated at 2.5 min intervals, which allowed stable release to be sustained over several hours. Mean [DA]_o_ evoked by a single electrical pulse in control conditions (in the presence of DHβE) across release sites was 1.13 ± 0.06 μm.

Light stimulation was delivered by an LED system (OptoLED; Cairn Research). DA release was evoked with full-field 470 nm blue light and a pulse duration of 2 ms. Experimental stimulation intensity was determined by delivering a light pulse sufficient to drive ∼50% maximal [DA]_o_. During recordings, slices were visualized on an upright microscope (Olympus BX50WI) with fluorescence optics for visualizing eYFP. Mean [DA]_o_ evoked by a single light pulse in control conditions was 0.90 ± 0.09 μm.

##### Experimental design and statistical analysis.

Data are represented as means ± SEM, and “*n*” refers to the number of experiments. Each experiment was performed at a single recording site in one brain slice. For each experiment at a given recording site, data for each variable were obtained in at least triplicate before averaging to obtain the value for that individual parameter. The number of animals in each dataset was ≥ 3. Data are expressed as extracellular concentration of dopamine ([DA]_o_) or as [DA]_o_ normalized to mean peak [DA]_o_ evoked by single pulses in control conditions. In all cases, [DA]_o_ displayed typical kinetics to peak and decay, indicative of good slice quality and were obtained from recording sites that maintained sufficiently stable levels of release over time. No data were excluded after acquisition.

Data acquired immediately before drug application were used as predrug control data and were compared with data acquired after drug effects had equilibrated after ∼10–20 min of application. Ratios for [DA]_o_ evoked by 5p/1p were obtained by dividing each 5p-evoked [DA]_o_ value with an average 1p-evoked [DA]_o_ value in the same condition at that recording site. In graphs representing peak [DA]_o_ evoked by 1p over time, missing data points were time points when data were acquired using different stimulation parameters interspersed among 1p stimulations. Comparisons for statistical significance were assessed by one- or two-way ANOVA, paired *t* tests, or Mann–Whitney *U* tests where data were not normally distributed using GraphPad Prism (RRID:SCR_002798).

##### Drugs.

DHβE, saclofen, and bicuculline were obtained from Tocris Bioscience. Baclofen, picrotoxin, and GABA were obtained from Sigma-Aldrich. Muscimol and CGP 55845 hydrochloride were obtained from Abcam. Stock aliquots of drugs were prepared at 1000–10,000× final concentrations in deionized water, aqueous acid (baclofen), or DMSO (picrotoxin) and stored at −20°C. DHβE was present throughout all experiments with electrical stimulation.

## Results

### GABA_A_ and GABA_B_ receptors inhibit striatal DA release

**Figure 1.**
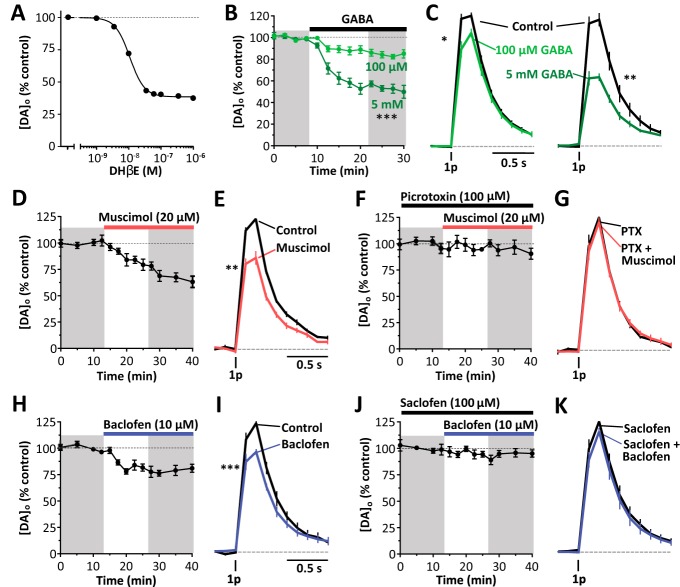
GABA, GABA_A_ agonist, or GABA_B_ agonists inhibit DA release. ***A***, Mean peak [DA]_o_ (± SEM) evoked by 1 electrical pulse versus applied DHβE concentration. Sigmoidal concentration–response curve fit, *R*^2^ = 0.93, *n* = 9. Shown is the mean peak [DA]_o_ (± SEM) versus time (***B***, ***D***, ***F***, ***H***, ***J***) and the mean [DA]_o_ (± SEM) versus time (***C***, ***E***, ***G***, ***I***, ***K***) evoked by 1 electrical pulse before and after application of: 100 μm GABA, *n* = 7, or 5 mm GABA, *n* = 5, ***p < 0.001, two-way ANOVA (***B***, ***C***); 20 μm muscimol, *n* = 5 (***D***, ***E***); 20 μm muscimol in the presence of 100 μm picrotoxin, *n* = 5 (***F***, ***G***); 10 μm baclofen, *n* = 7 (***H***, ***I***); or 10 μm baclofen in the presence of 100 μm saclofen, *n* = 5 (***J***, ***K***). Data are normalized to peak [DA]_o_ before drug application. Shaded areas are used to obtain illustrated data and statistical comparisons. *p < 0.05, **p < 0.01, ***p < 0.001, Mann–Whitney tests. The nAChR antagonist DHβE (1 μm) is present throughout.

We assessed whether GABA can modulate striatal DA release in the absence of nAChR activity using the antagonist DHβE (1 μm) to inhibit nAChRs as described previously (Rice and Cragg, 2004; Threlfell et al., 2012). We first confirmed that the DHβE concentration was supramaximal for inhibition of DA release by confirming the concentration–response relationship for DHβE (1 nm to 1 μm) on [DA]_o_ evoked by a single electrical pulse. DHβE concentration-dependently inhibited evoked [DA]_o_ (Fig. 1*A*; sigmoidal concentration–response curves, *R*^2^ = 0.93; Hill slope, −1.92, IC_50_, 11 nm, *n* = 9) and 1 μm DHβE was supramaximal for inhibition of [DA]_o_.

GABA was applied to striatal slices in the presence of the nAChR antagonist DHβE (1 μm). Bath application of GABA (100 μm, 5 mm) concentration dependently reduced DA release evoked by single electrical pulses by ∼15–50% (Fig. 1*B*,*C*; two-way ANOVA: *F*_(1,10)_ = 48.32, *p* < 0.0001, 100 μm GABA, *U* = 0, *p* = 0.016, *n* = 7; *U* = 6, 5 mm, *p* = 0.0079, *n* = 5, Mann–Whitney tests), indicating that GABA is capable of inhibiting DA release independently from any effects on ChI input to nAChRs.

To identify which GABA receptors can inhibit DA release, we tested the effect of GABA_A_ or GABA_B_ agonists. Activation of GABA_A_ receptors with muscimol (20 μm) suppressed DA release evoked by single electrical pulses by ∼30% (Fig. 1*D*,*E*: *U* = 0, *p* = 0.0079, *n* = 5, Mann–Whitney test) and this effect was prevented by prior application of GABA_A_ channel blocker picrotoxin (100 μM), confirming an effect via GABA_A_ receptors (Fig. 1*F*,*G*: *U* = 11, *p* = 0.85, *n* = 5, Mann–Whitney test). Activation of GABA_B_ receptors with baclofen (10 μm) also suppressed DA release evoked by single electrical pulses by ∼25% (Fig. 1*H*,*I*: *U* = 0, *p* = 0.0006, *n* = 7, Mann–Whitney test) and this effect was confirmed to be due to GABA_B_ receptors by prior application of the GABA_B_ antagonist saclofen (100 μM), which prevented the effect of baclofen (Fig. 1*J*,*K*: *U* = 5, *p* = 0.11, *n* = 5, Mann–Whitney test).

### GABA receptors on frequency sensitivity of DA release

**Figure 2.**
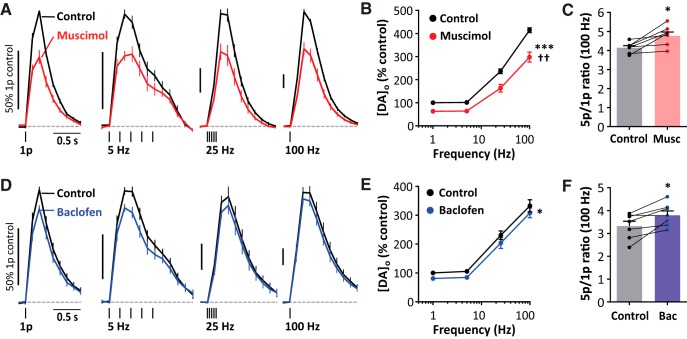
GABA_A_ and GABA_B_ receptor agonists modify DA release during pulse trains. Shown is mean [DA]_o_ (± SEM) versus time (***A***,***D***) and mean peak [DA]_o_ (± SEM) versus stimulation frequency (***B***,***E***) for 1- or 5-pulse trains in control conditions (black); 20 μm muscimol, *n* = 7 (***A***,***B***) (red); or 10 μm baclofen (***D***,***E***) (blue); two-way ANOVA, effect of drug *p < 0.05, ***p < 0.001, drug × frequency interaction ^††^*p* < 0.01. Data are normalized to peak [DA]_o_ before drug application. ***C***, ***F***, Ratio of peak [DA]_o_ released by 5p versus 1p (100 Hz) in control conditions, muscimol (***C***), or baclofen (***F***). *p < 0.05, paired *t* tests versus control. The nAChR antagonist DHβE (1 μm) is present throughout.

**Figure 3.**
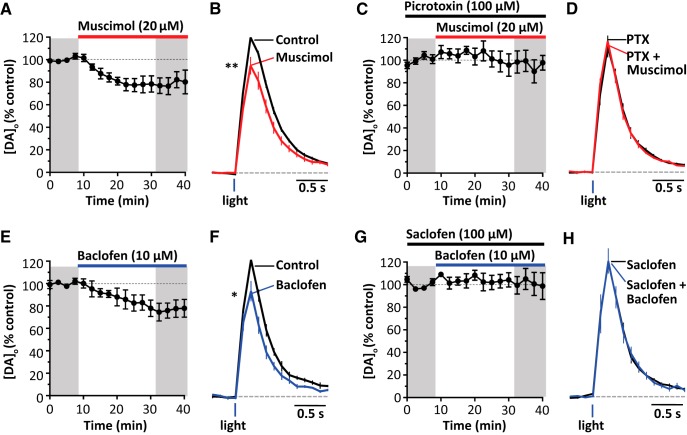
GABA receptors inhibit optogenetically stimulated DA release. Shown is mean peak [DA]_o_ (± SEM) versus time (***A***,***C***,***E***,***G***) and mean [DA]_o_ (± SEM) versus time (***B***,***D***,***F***,***H***) evoked by 1 light pulse before and during application of 20 μm muscimol, *n* = 5 (***A***,***B***); 20 μm muscimol in the presence of 100 μm picrotoxin, *n* = 5 (***C***,***D***); 10 μm baclofen, *n* = 4 (***E***,***F***); or 10 μm baclofen in the presence of 100 μm saclofen, *n* = 6 (***G***,***H***). Data are normalized to mean peak [DA]_o_ before drug application. *p < 0.05, **p < 0.01, Mann–Whitney tests.

We investigated whether GABA receptor activation inhibited DA release during trains of stimuli and across a range of frequencies. Muscimol significantly decreased [DA]_o_ evoked by 5-pulse trains at all frequencies tested (Fig. 2*A*,*B*; two-way ANOVA: drug effect: *F*_(1,48)_ = 67.55; *p* = 0.0025, frequency effect: *F*_(3,48)_ = 259.6, *p* < 0.0001, *n* = 7) and, furthermore, there was a significant interaction between stimulation frequency and drug effect (Fig. 2*B*; two-way ANOVA: *F*_(3,48)_ = 5.485, *p* = 0.0025), which was borne out by an increase in the 5p/1p ratio examined at 100 Hz (Fig. 3*C*; paired *t* test: *t*_(6)_ = 3.46, *p* = 0.014). In other words, activation of GABA_A_ receptors can marginally promote the contrast in DA signals released by different firing patterns or rate.

Baclofen significantly decreased evoked [DA]_o_ (Fig. 2*E*; two-way ANOVA: *F*_(1,48)_ = 6.271, drug effect: *p* = 0.016; frequency effect: *F*_(3,48)_ = 144.5, *p* < 0.0001, *n* = 7). We did not detect a significant interaction between stimulation frequency and drug effect (Fig. 2*E*: two-way ANOVA: *F*_(3,48)_ = 0.040, *p* = 0.99); however, the 5p/1p ratio for [DA]_o_ evoked at 100 Hz was slightly increased with baclofen (Fig. 2*F*: paired *t* test: *t*_(6)_ = 3.272, *p* = 0.017), suggesting that GABA_B_ receptors only marginally change the contrast in DA signals released by different activity.

### GABA receptors inhibit DA release evoked by targeted optogenetic stimulation

To determine whether the inhibition of DA release by GABA receptors depended on the coactivation during electrical stimulation of some other local neuron type or input, we used an optogenetic approach to selectively activate DA axons only. We expressed ChR2-eYFP in DA neurons and axons in DAT-Cre mice using an established viral approach as described previously (Threlfell et al., 2012; Brimblecombe and Cragg, 2015). Either the GABA_A_ agonist muscimol or the GABA_B_ agonist baclofen suppressed DA release evoked by single blue light pulses (muscimol, Fig. 3*A*,*B*; *U* = 0, *p* = 0.0079, *n* = 5; Mann–Whitney test; baclofen, Fig. 3*C*,*D*; *U* = 0, *p* = 0.029, *n* = 4, Mann–Whitney test), indicating that GABA receptor agonists do not require coincident activation of another striatal input to suppress DA release. We also confirmed that, as with electrical stimulation, the effects of muscimol and baclofen were prevented by prior application of antagonists for respectively GABA_A_ (picrotoxin, Fig. 3*C*,*D*; effect of agonist, *p* = 0.36, *n* = 6, Mann–Whitney test, *U* = 12) or GABA_B_ receptors (saclofen, Fig. 3*G*,*H*; effects of agonist, *p* = 0.127, *n* = 5, Mann–Whitney test, *U* = 5).

### GABA operates a tonic inhibition on DA release

Some striatal GABA neurons are tonically active and it has been reported that there is an ambient GABA tone in striatum (Ade et al., 2008; Kirmse et al., 2008, 2009; Santhakumar et al., 2010; Cepeda et al., 2013). We therefore tested whether there was tonic inhibition of DA release by exploring the effects of GABA receptor antagonists on DA release evoked either optogenetically or electrically.

**Figure 4.**
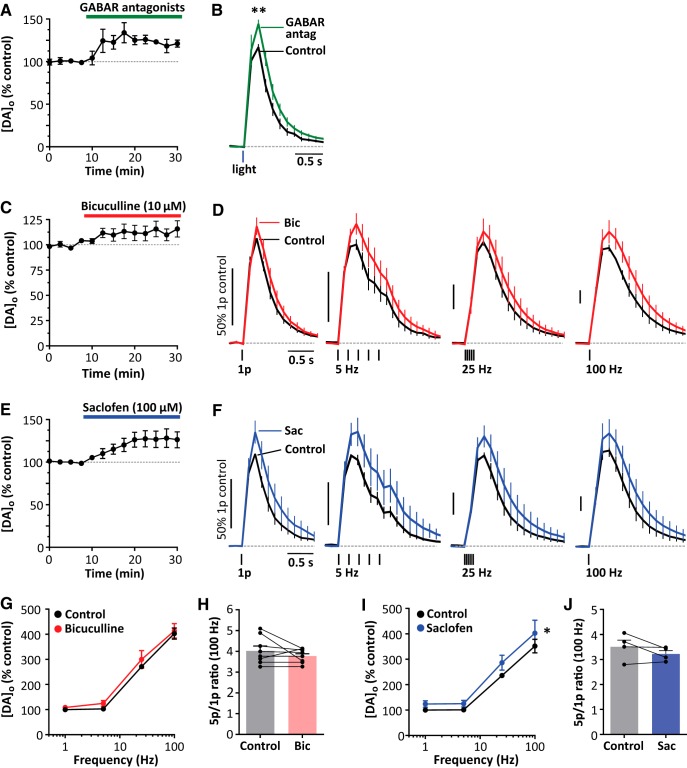
GABA receptor antagonists increase DA release. ***A***, ***B***, Mean peak [DA]_o_ (± SEM) versus time (***A***) and mean [DA]_o_ (± SEM) versus time (***B***) evoked by 1 light pulse before and in the presence of 3.5 μm CGP 55845 and 10 μm bicuculline (GABAR antagonists). **p < 0.01, Mann–Whitney test. Shown is the mean ***C***, ***E***, Mean peak [DA]_o_ evoked by 1 electrical pulse with the application of 10 μm bicuculline (***C***) or 100 μm saclofen (***E***), all in the presence of 1 μm DHβE. ***D***, ***F***, Mean [DA]_o_ (± SEM) versus time in control conditions (black), 10 μm bicuculline (***D***) (red), or 100 μm saclofen (***F***) (blue). ***G***, ***I***, Mean peak [DA]_o_ (± SEM) versus stimulation frequency in control conditions (black), with bicuculline (***G***), or with saclofen (***I***). Data are normalized to peak 1p-evoked [DA]_o_ before drug application; two-way ANOVA, effect of drug *p < 0.05. ***H***, ***J***, Ratio of peak [DA]_o_ released by 5p versus 1p (100 Hz) in control conditions, bicuculline (***H***), or with saclofen (***J***).

We found that coapplication of GABA_A_ and GABA_B_ antagonists bicuculline (10 μm) and CGP 55845 (2 μm), respectively, significantly enhanced [DA]_o_ evoked by single light pulses in DAT-Cre ChR2-expressing mice by ∼20% (Fig. 4*A*,*B*; *p* = 0.0079, *n* = 5, Mann–Whitney test, *U* = 0), indicating a tonic inhibition of DA release by endogenous striatal GABA in the absence of other stimuli. We investigated which receptors could mediate inhibition of DA release by endogenous GABA using electrical stimulation in wild-type mice (in the presence of DHβE). GABA_A_ antagonist bicuculline appeared to slightly elevate, but did not significantly increase, evoked [DA]_o_ (Fig. 4*C*,*D*,*G*; two-way ANOVA: effect of drug, *F*_(1,44)_ = 1.58, *p* = 0.215; effects of frequency: *F*_(3,44)_ = 128.4, *p* < 0.0001, *n* = 5) or interact with stimulation frequency (Fig. 4*C*: two-way ANOVA: *F*_(3,44)_ = 0.115, *p* = 0.95) or the 5p:1p ratio (Fig. 4*H*: *t*_(7)_ = 1.217, *p* = 0.263, paired *t* test). The GABA_B_ antagonist saclofen significantly increased evoked [DA]_o_ (Fig. 4*E*,*F*,*I*; two-way ANOVA: effect of drug, *F*_(1,24)_ = 4.87, *p* = 0.0371; effects of frequency, *F*_(3,24)_ = 57.8, *p* < 0.0001). There was no significant interaction with stimulation frequency (Fig. 4*C*: two-way ANOVA: *F*_(3,24)_ = 0.205, *p* = 0.89) or 5p:1p ratio (Fig. 4*J*, *t*_(3)_ = 1.49, *p* = 0.233, paired *t* test).

## Discussion

Here, we show that GABA, GABA_A_ receptors, and GABA_B_ receptors in dorsal striatum inhibit DA release and that these actions are not mediated via regulation of striatal ACh acting at nAChRs. By eliminating ChIs as a necessary site of action of GABA, a direct action on DA axons becomes plausible as a site for GABA action on DA transmission. Furthermore, we show that GABA receptor antagonists increase DA release evoked by single optogenetic stimuli, revealing that there is tonic inhibition of DA release by endogenous striatal GABA arising from a tonically active source or ambient level.

### GABA receptor influence on DA output does not require striatal ACh

We found that both GABA_A_ and GABA_B_ receptor ligands can inhibit DA release whether evoked electrically in the presence of nAChR antagonist or optogenetically by targeted activation of ChR2-expressing DA axons in DAT-Cre mice. Light-activated DA release is not under tonic control by striatal nAChRs in these stimulation conditions (Threlfell et al., 2012; Melchior et al., 2015). Because GABA receptor effects were seen in the absence of nAChR activation, they are unlikely to require GABA receptors on ChIs. These findings are consistent with a study in NAc indicating that GABA_B_-mediated control of DA is independent of ACh input (Pitman et al., 2014). We note that muscarinic M5 receptors on DA axons have been suggested in some studies to regulate DA release (Bendor et al., 2010; Shin et al., 2015), but we found that electrically evoked DA release in the presence of DHβE and optogenetically evoked DA release were not attenuated by muscarinic receptor antagonists (Threlfell et al., 2010, 2012), so these effects of GABA and GABA receptors are unlikely to be mediated by alternative actions of ACh at M5 mAChRs on DA axons.

Activation of GABA receptors had limited effects on the frequency sensitivity of evoked DA release. Activation of GABA_A_ receptors, but not GABA_B_ receptors, interacted in a statistically significant manner with frequency and number of stimulus pulses to slightly promote the ratio of [DA]_o_ evoked by high-frequency trains over single pulse release, but the effect size was modest. Therefore, GABA receptor activation primarily limits the overall amplitude of DA output with only a minor additional enhancement in the frequency filtering, unlike ACh, which profoundly changes the relationship of DA output to presynaptic activity (Rice and Cragg, 2004; Zhang and Sulzer, 2004).

### Tonic inhibition

We found that GABA receptor antagonists can enhance DA release evoked by a short single optogenetic stimulation of ChR2-expressing DA axons in DAT-Cre mice, suggesting that DA release is under tonic inhibition by GABA. Light activation is targeted to DA axons and should not activate GABA release from other striatal neurons. Furthermore, the stimulus is sufficiently short (2 ms) that evoked DA release should not be under the control of any GABA that might be coreleased by this stimulus. Mesostriatal DA neurons can apparently synthesize, store, and corelease GABA (Tritsch et al., 2014; Kim et al., 2015), which can evoke inhibitory currents in postsynaptic medium spiny neurons, but it is unlikely that any GABA coreleased with DA could simultaneously gate the concurrent release of DA evoked by the same single 2 ms stimulus. Rather, it is more likely that DA release is under tonic inhibition by a preexisting striatal GABA tone. A tonic inhibition is consistent with previous reports that there is an ambient GABA tone in striatum detected as a GABA_A_ receptor-mediated current in postsynaptic neurons even in *ex vivo* preparations (Ade et al., 2008; Kirmse et al., 2008, 2009; Santhakumar et al., 2010; Cepeda et al., 2013). Our findings indicate that tonic inhibition of DA release can readily be detected, at least for GABA_B_ receptors.

An ambient GABA tone that can act on DA axons to limit DA output should not be entirely surprising. At least one type of GABAergic interneuron, the LTS interneuron, is capable of autonomous firing in striatal slices (Beatty et al., 2012). Furthermore, striatal neurons are ∼95% GABAergic, so even low levels of GABA release per neuron could potentially summate for significant impact. No GABAergic axoaxonic synapses have been identified on DA axons, but this need not preclude an interaction. GABA can spillover for extrasynaptic actions in many neurons (Farrant and Nusser, 2005) and might act on DA axons through extrasynaptic effects. The volume of striatum reached by the extensive axonal arbor of a single DA neuron (Matsuda et al., 2009) can be calculated from striatal neuron counts (Oorschot, 1996) to encompass ∼70,000 GABAergic neurons (plus additional non-neuronal cells that might provide a source of GABA). This large number of potential GABA sources might readily spillover and/or otherwise summate to provide an ambient GABA tone that can act at GABA receptors to limit DA output. Striatal GABA might therefore be in a position to influence striatal output, not only though direct actions on output neurons and local interneuron and input networks, but also by governing DA transmission.

### Direct versus indirect actions of GABA receptors

The site of GABA receptor localization remains undefined. DA neurons in substantia nigra express GABA_A_ and GABA_B_ receptors and these are certainly functional in somatodendritic compartments (Bowery et al., 1987; Nicholson et al., 1992; Boyes and Bolam, 2003; Brazhnik et al., 2008), but their trafficking and localization to DA axons has not yet been demonstrated. GABA_B_ receptors have been immunocytochemically detected in striatal neuropil, consistent with localization of these receptors on DA axons (Charara et al., 2000), but GABA_A_ receptors have not yet been shown on DA axons. It can be difficult to verify the ultrastructural location of membrane bound receptors using classic immunocytochemical methods, which depend on appropriate fixation methods and conditions for antibody penetration; absence of proof is not proof of absence.

Key candidates for alternative sites of action can however be eliminated. Here, we exclude actions of GABA receptors on ChIs acting through downstream nAChRs. Furthermore, GABAergic interneurons are unlikely as intermediaries: for example, if GABA_A_ receptors are acting indirectly through an intermediary GABA circuit that modulates DA via downstream GABA_B_ receptors, then GABA_A_ receptor agonists would be expected to inhibit the intermediary GABA circuit and therefore increase DA release, which is the opposite of what was seen here. The remaining candidate locations for the GABA receptors that regulate DA are therefore either an undisclosed neuron or other cell type with currently unknown actions on DA or, more parsimoniously, DA axons themselves. Responses to GABA have been described for axons of several other neurons (Kullmann et al., 2005; Trigo et al., 2008; Bucher and Goaillard, 2011). For example, in other CNS neurons, GABA_A_ receptors can reduce axonal spike amplitude and propagation and promote spike failures (Zhang and Jackson, 1995; Ruiz et al., 2003; Verdier et al., 2003), whereas GABA_B_ receptors inhibit voltage-gated Ca^2+^ channels (Sun and Chiu, 1999). Similar mechanisms could account for GABAergic inhibition of DA release from DA axonal arbors.

In conclusion, we show here not only that GABA_A_ and GABA_B_ receptors can gate DA output, but that there is a tonic inhibition by endogenous GABA through an apparent ambient GABA tone. In addition to directly regulating striatal output, striatal GABA tone might therefore also govern striatal integration via dampening DA output. We exclude cholinergic interneurons acting through nAChRs on DA axons as intermediaries for GABA regulation of DA, thereby adding support to the hypothesis that GABA is acting directly on DA axons. With the assistance of new genetic tagging and imaging tools, future studies should revisit the potential localization of GABA_A_ and GABA_B_ receptors to DA axons.
